# Surface micropattern limits bacterial contamination

**DOI:** 10.1186/2047-2994-3-28

**Published:** 2014-09-17

**Authors:** Ethan E Mann, Dipankar Manna, Michael R Mettetal, Rhea M May, Elisa M Dannemiller, Kenneth K Chung, Anthony B Brennan, Shravanthi T Reddy

**Affiliations:** 1 Sharklet Technologies, Inc, 12635 E. Montview Blvd, Suite 160, Aurora, CO 80045, USA; 2 Department of Materials Science and Engineering, University of Florida, Gainesville, FL, USA

## Abstract

**Background:**

Bacterial surface contamination contributes to transmission of nosocomial infections. Chemical cleansers used to control surface contamination are often toxic and incorrectly implemented. Additional non-toxic strategies should be combined with regular cleanings to mitigate risks of human error and further decrease rates of nosocomial infections. The Sharklet micropattern (MP), inspired by shark skin, is an effective tool for reducing bacterial load on surfaces without toxic additives. The studies presented here were carried out to investigate the MP surfaces capability to reduce colonization of methicillin-sensitive *Staphylococcus aureus* (MSSA) and methicillin-resistant *S. aureus* (MRSA) compared to smooth control surfaces.

**Methods:**

The MP and smooth surfaces produced in acrylic film were compared for remaining bacterial contamination and colonization following inoculation. Direct sampling of surfaces was carried out after inoculation by immersion, spray, and/or touch methods. Ultimately, a combination assay was developed to assess bacterial contamination after touch transfer inoculation combined with drying (persistence) to mimic common environmental contamination scenarios in the clinic or hospital environment. The combination transfer and persistence assay was then used to test antimicrobial copper beside the MP for the ability to reduce MSSA and MRSA challenge.

**Results:**

The MP reduced bacterial contamination with log reductions ranging from 87-99% (LR = 0.90-2.18; *p* < 0.05) compared to smooth control surfaces. The MP was more effective than the 99.9% pure copper alloy C11000 at reducing surface contamination of *S. aureus* (MSSA and MRSA) through transfer and persistence of bacteria. The MP reduced MSSA by as much as 97% (LR = 1.54; *p* < 0.01) and MRSA by as much as 94% (LR = 1.26; *p* < 0.005) compared to smooth controls. Antimicrobial copper had no significant effect on MSSA contamination, but reduced MRSA contamination by 80% (LR = 0.70; *p* < 0.005).

**Conclusion:**

The assays developed in this study mimic hospital environmental contamination events to demonstrate the performance of a MP to limit contamination under multiple conditions. Antimicrobial copper has been implemented in hospital room studies to evaluate its impact on nosocomial infections and a decrease in HAI rate was shown. Similar implementation of the MP has potential to reduce the incidence of HAIs although future clinical studies will be necessary to validate the MP’s true impact.

## Background

Environmental surface contamination provides a potential reservoir for pathogens to persist and cause infection in susceptible patients [1,2]. Environmental surfaces near patients such as bed rails, tray tables, telephones, bedside tables, patient chairs, and nurse call buttons are often heavily contaminated [3-6]. Methicillin-resistant *Staphylococcus aureus* (MRSA) and Vancomycin-resistant *Enterococcus* (VRE) have been shown to survive on inanimate surfaces for a minimum of a few weeks and in some cases months [1,7-11]. Pathogens contaminate surfaces through large volume surface soaking (e.g. spills) or micro-droplet aspirations (e.g. sneezes) and are transferred subsequently to healthcare workers’ hands and other objects (e.g. touch events) [12-14]. Recent evidence confirms that patients admitted to rooms previously occupied by patients infected or colonized with MRSA or VRE have an increased risk of acquiring the same pathogen as the prior room occupants [15-20].

Healthcare infection control guidelines from the Centers for Disease Control and Prevention (CDC) now emphasize the importance of cleaning and disinfecting “high-touch surfaces” and monitoring these activities to maintain a sanitary environment in the hospital [21]. These documents reflect an evolving mindset that patient area environmental cleanliness in healthcare settings plays a significant role in infection prevention and control. Despite the increased attention to environmental hygiene, recent studies have shown that as few as 40% of near patient surfaces are being cleaned in accordance with existing hospital policies [6,10,16]. Surprisingly few technological surface improvements have been implemented to address the problem of contaminated surfaces that exist between terminal cleanings [22]. Among the few, antimicrobial copper has recently been implemented as a technology to prevent surface contamination between cleanings. In one copper trial, patients who developed HAI and/or colonization of MRSA and VRE was significantly reduced from 0.123 to 0.071 (*p* = 0.02) for patients that resided in ICU rooms with copper as opposed to ICU rooms without copper [23]. The proportion of patients developing HAI alone was reduced from 0.081 to 0.034 (*p* = 0.013) where copper was used [23]. Unfortunately, copper and antimicrobial silver are expensive to implement and both utilize kill mechanisms which have potential to select for resistant organisms [24]. While the results of the copper trials may require further validation [25,26], the data from these studies indicate that sustained surface contamination reduction may offer a clinical benefit.

**Figure 1:**
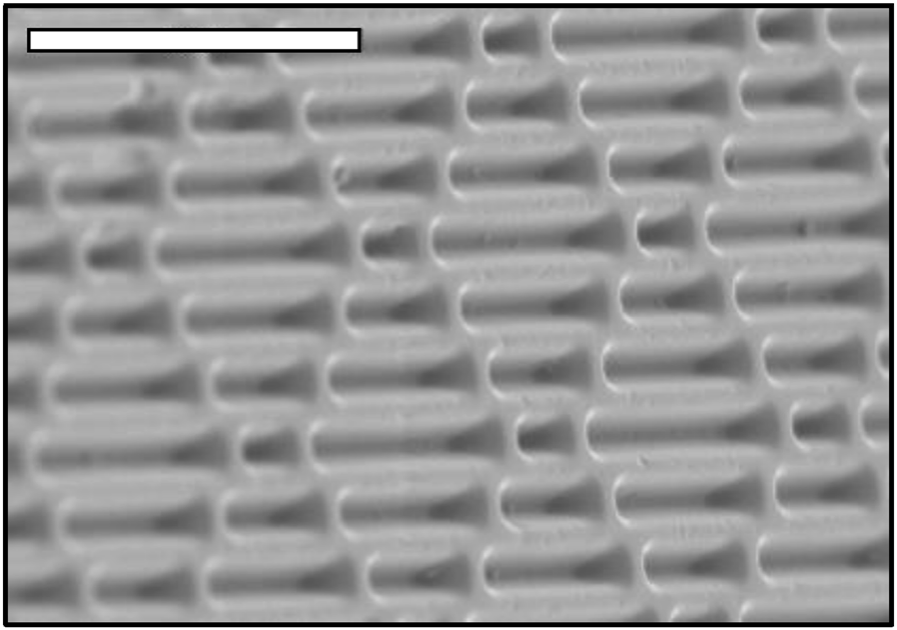
**Scanning electron micrograph of Sharklet micro-patterned (MP) acrylic material.** The scale bar in the micrograph represents 20 μm.

A micropattern (MP) surface was evaluated to address the need for improved surface technology to resist bacterial contamination. Specifically, previous studies show the Sharklet MP to be the most effective among ordered topographies (pillars, channels, other geometries) for inhibiting bioadhesion (Figure 1) [27,28]. The MP reduces colonization of a variety of marine organisms and human pathogens in nutrient-rich environments [27,29-34]. The MP is a physical surface modification that does not introduce chemical additives or antimicrobials; therefore the bulk properties of the material are not affected by the presence of the textured surface. Alternative surface modifications reporting to limit bacterial contamination exist but were not tested here. They include examples like photo-activating agents, polyethylene glycol, and diamond-like carbon films and were reviewed recently for their roles in contamination mitigation [35].

Demonstration of MP efficacy requires testing designed to assess the remaining viable bacteria directly from the surface rather than enumerating bacteria remaining in solutions exposed to the surface as done in existing standardized protocols for assessing antimicrobial surfaces [36]. Specific bacterial contamination scenarios were examined using immersion, spray, or touch transference inoculation methods with, MSSA and MRSA to mimic common contamination scenarios. Ultimately, through validation of individual inoculation methods, a combination of both bacterial transfer and persistence events were combined into a single assay. The combination method was used to compare the reduction of MSSA and MRSA on the MP to antimicrobial copper.

## Methods

### Bacterial strains, growth conditions, and inoculums

Bacterial strains used for testing included methicillin-sensitive *Staphylococcus aureus* (MSSA; ATCC 6538) or methicillin-resistant *S. aureus* (MRSA; ATCC 43300), Each were grown in a shaking incubator at 37°C for 18–24 h in tryptic soy broth (TSB) media (Hardy Diagnostics, Santa Maria, CA). Prior to inoculation, strains were sub-cultured into fresh TSB at 1:100 dilution and grown for 4 h. Inoculum suspensions were prepared by pelleting, re-suspending and adjusting the cell concentration of the broth cultures in phosphate buffered saline (PBS; Hardy Diagnostics, Santa Maria, CA) using a known OD_600_/CFU ratio. Following the experiment, bacterial inoculation suspensions were evaluated for CFUs. Experiments with inoculums within 0.5 log units of the target inoculum were accepted.

### Bacterial challenge on surfaces

#### ***Test surface materials***

Flat polydimethylsiloxane elastomer (PDMSe; Dow Corning, Midland, MI) or acrylic film (Flexcon, Spenser, MA), was either cast against nickel shims or embossed with an inverse Sharklet™ micropattern (MP) or smooth surface (controls) by Sharklet Technologies, Inc (STI; Aurora, CO). The inverse MP consists of 2 μm wide rectangular features with nonadjacent repeating lengths that are recessed into the surface and arranged in a periodic diamond pattern with 2 μm spacing and a feature depth of 3 μm (Figure 1). Copper foil, a 99.9% pure alloy C11000 (Alaskan Copper and Brass Company, Seattle, WA), registered as a US EPA antimicrobial was purchased for antimicrobial copper testing. Each elastomer, plastic, or copper foil sample was firmly adhered to the bottom of a Petri dish, sterilized for 10 m with 95% ethanol, rinsed 3 times with deionized water and allowed to dry prior to each experiment. In each experimental method discussed, un-patterned smooth surfaces of the identical plastic material, or smooth acrylic for antimicrobial copper comparisons, were used as standards to achieve percent reduction calculations.

### Surface testing methods

Experimental methods used to evaluate attachment and survival were based on a review of previous studies assessing surface contamination or antimicrobial efficacy on surfaces [1,5,7,8,36-41]. Three assays were developed that each evaluated varied aspects of real-world surface contamination scenarios.

#### ***Spray inoculation assay***

To achieve even bacterial loading, the spray inoculation method was used to test MSSA. Suspensions ranging from 1 × 10^5^ to 1 × 10^7^CFU/ml were prepared from log-phase growth cultures in sterile 1 × PBS. A Central Pneumatic Professional gravity-fed paint sprayer (Harbor Freight Tools, Camarillo, CA) was sterilized by spraying 50 ml of 95% ethanol through the device and rinsed with 100 ml of sterile deionized water. Using sterile 1 × PBS, the appropriate spray conditions were optimized to deposit between 100–200 μl of fluid per dish. Per spray event, 5–6 plates were secured in the biological safety cabinet on test tube racks angled at approximately 45 degrees. The sprayer was connected to compressed nitrogen tank (General Air, Denver, CO) and was loaded with 50–100 ml of prepared bacterial suspension. Test and control surfaces were cut into 40 mm radius semi-circles and placed side-by-side in a single Petri dish. Experimental plates were weighed before and after spraying and the volume of delivered inoculum was calculated to ensure the samples were within the appropriate spray inoculum range for enumeration through RODAC sampling. RODAC sampling occurred directly following drying of 30 m at ambient conditions without rinsing. Additionally, a disruption and dilution sampling method, as described below as a previously optimized MSSA quantification standard protocol, was used as supplementary quantification methods to confirm the ability for the RODAC plates to recover cells from the surface after spray inoculation.

#### ***Immersion inoculation assay***

Bacterial inoculums of MSSA or MRSA ranging from 1 × 10^3^ to 1 × 10^4^ CFU/ml completely submerged the test samples in the dish for 1 h at room temperature (RT). The bacterial suspension was then removed and the dishes were rinsed with sterile 1 × PBS 3 times, for 10 s while rotating at 80 rpm, to remove non-attached cells. After discarding the final rinsate, surfaces were dried under ambient conditions for 1 h then sampled for viable bacteria using RODAC contact plates as described below.

#### ***Touch transference inoculation assay***

MSSA and MRSA were used to evaluate the performance of the MP in this assay. Test and control surfaces were cut into 40 mm radius semi-circles and placed side-by-side in a single Petri dish. Bacterial suspensions (5 ml) ranging from 1 × 10^5^ to 1 × 10^7^ CFU/ml were used to flood sterile velveteen cloths (Bel-Art Products, Wayne, NJ) that lined the bottom of sterile Petri dishes [42]. Sterile velveteen cloth was placed on a replica plating tool (Bel-Art Products, Wayne, NJ) and inverted onto saturated velveteen-containing bacterial inoculum for 10 s before being placed onto test and control surfaces for a 10 s contact time. The test surfaces were then allowed to dry for 5–10 s under ambient conditions before being sampled using RODAC contact plates, as described below.

#### ***Combination transference and persistence assay***

Suspensions of MSSA and MRSA organisms ranging from 1 × 10^5^ to 1 × 10^7^ CFU/ml were each used to challenge smooth and the MP acrylic films. Test and control acrylic film was cut into 40 mm radius semi-circles and placed side-by-side in a single Petri dish. Touch transference inoculation (described above) was used to inoculate the challenge surfaces. Reduction of touch transference was measured after 0 m of drying and reduction of persistence was measured after 90 m of drying was added in combination while each time point was sampled using RODAC plates. Similarly, antimicrobial copper was subjected to challenge with MSSA and MRSA using the same combination transference and persistence testing in head-to-head comparison with the MP acrylic film.

### Sampling bacterial load

#### ***RODAC contact agar***

Following inoculation and processing of each sample surface, bacterial load was quantified using RODAC contact agar plates. In each test, per organism investigated, the inoculation range was determined experimentally as being optimal for yielding a countable range of colonies on RODAC contact plates (BBL Prepared RODAC Plate, Trypticase Soy Agar with Lecithin and Polysorbate 80, BD, Franklin Lakes, NJ), which were used for cell recovery from sample surfaces. The agar contact method was used to directly quantify bacterial CFU transferred from the surface to a 60 mm diameter RODAC contact plate for enumeration of total colony counts. The RODAC contact plates were pressed onto inoculated surfaces for 5 s while avoiding air bubbles between the surface and RODAC plate. The RODAC plates were then incubated for 18–24 h at 37°C. The RODAC plates were photographed and counted using magnification and Image J colony counting methods. The resulting colonies were enumerated, log transformed, and recorded as log CFU/RODAC.

#### Disruption and dilution plating

Sterile biopsy punches (4 mm; VWR International, Radnor, PA) were used to obtain samples from inoculated surfaces. Punches were dropped into conical tubes, each containing 1 ml of fresh Dey-Engley (DE) neutralization buffer (Sigma, St. Louis, MO). The tubes were sonicated for two minutes with 30 s vortexes before and after sonication [41]. Serial dilution of the eluted bacteria in DE buffer was then plated onto TSA, and the plates were incubated for 18–24 h at 37°C. Resulting colonies were enumerated, log transformed, and recorded as log CFU/ml.

### Microscopy analysis

Scanning electron microscopy was used to visualize MSSA due to its ability to potentially exist within the patterned surface. After bacterial immersion, two samples of each the MP and two smooth samples were retained for analysis without RODAC exposure, while another two samples of each surface were stamped with a RODAC plate. Each sample was subsequently fixed with osmium tetroxide gas (Electron Microscopy Sciences, 19150) for 45 m and then subjected to a dehydration series with 10 m incubations in 25, 50, 75, and finally 95% ethanol (Decon Labs Inc, King of Prussia, PA), then air-dried overnight at ambient conditions. Two 8 mm circles were taken near the center of each sample with a biopsy punch (Fisher, Waltham, MA), mounted, sputter-coated with gold, and analyzed via SEM. Two images were taken per smooth sample and four images were taken per the MP sample for qualitative image analysis.

### Data reporting and statistical analysis

Each experiment consisted of at least three experimental replicates per surface type (the MP and smooth control), generating a log reduction value calculated from paired comparison of the MP and smooth surface mean log cell densities. Each single experiment was repeated at least three times to generate least squares mean log reduction (LR) values and establish statistical significance [43]. The resulting log reductions were subjected to a single t-Test and, when appropriate, an ANOVA analysis with a Tukey test to generate statistical significance and grouping across samples, materials, and strain types. Smooth control surface log cell densities were compared where appropriate to determine experimental variance for establishing optimum assay conditions.

## Results

### Bacterial attachment to surfaces

**Figure 2:**
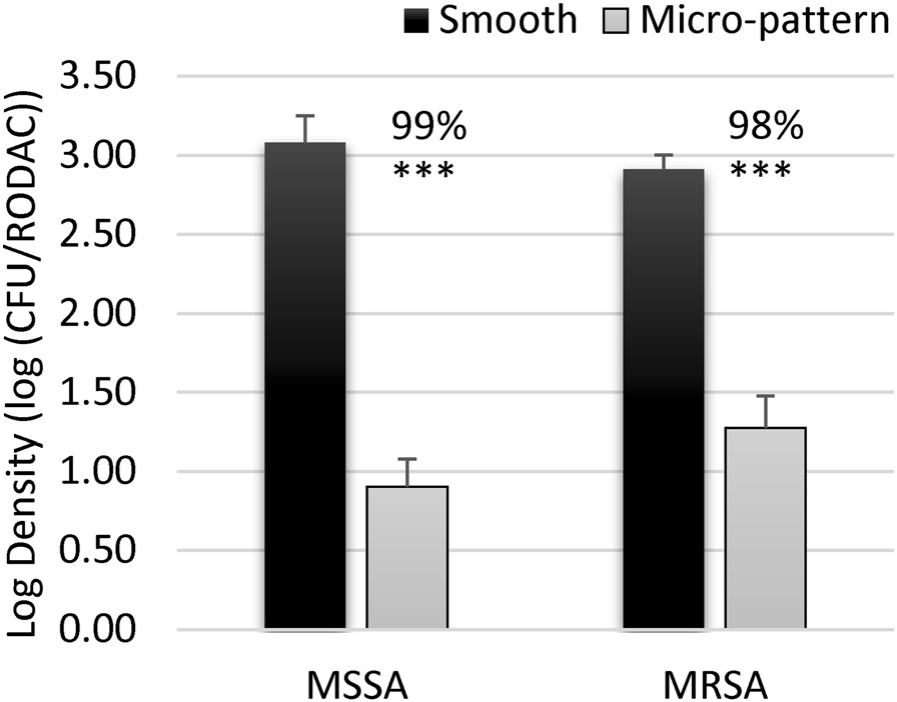
**Microbial attachment.** MSSA and MRSA were incubated in suspension on smooth (black bars) or micro-patterned (grey bars) acrylic film for 1 h. After rinsing 3 times and drying for 1 h the remaining viable bacteria on the surfaces were quantified. The plot represents average log densities and standard error of the mean. Significance was determined using a single t-Test of the log reduction data points. The average log reduction values were then used to calculate the median percent reduction values indicated above each column. *p <* 0.005 (***).

The immersion assay with RODAC recovery was used to quantify bacterial attachment to representative acrylic The MP and smooth surfaces after being inoculated with a bacterial suspension. MSSA and MRSA demonstrated significantly reduced attachment to the MP surfaces compared to smooth controls, with 99% (LR = 2.18; *p* < 0.001) and 98% (LR = 1.64; *p* < 0.001) reductions of each of these organisms, respectively (Figure 2).

### Bacterial persistence on surfaces

**Figure 3:**
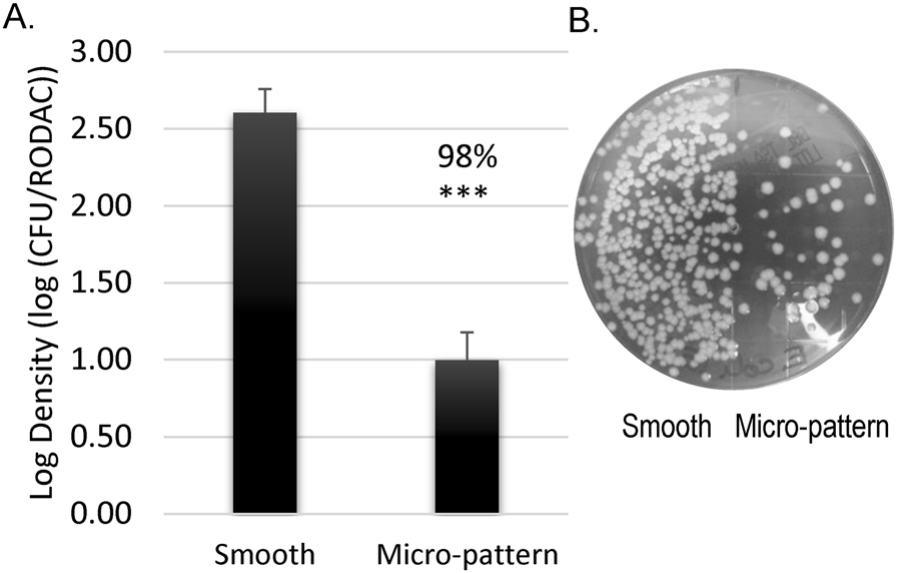
**Microbial persistence.** Smooth and micro-patterned (MP) acrylic films were challenged with a sprayed inoculum and dried for 30 m. **A**.) Log densities of bacteria present on the surfaces of the MP compared to smooth controls for MSSA are plotted with the associated standard error of the mean. **B**.) A representative image of a RODAC contact plate after MSSA sampling, the MP surface (right) has fewer bacteria compared to the smooth surface (left). *p <* 0.005 (***) n = 3.

*S. aureus* (MSSA) was tested for persistence on the MP with RODAC recovery after a uniform spray inoculation technique, mimicking a common surface contamination event. MSSA was reduced by 98% (LR = 1.61; *p* < 0.005) on the MP compared to smooth controls (Figure 3A). The MP reduction of MSSA contamination was also visually apparent using RODAC recovery (Figure 3B). Sprayed surfaces were also sampled using disruption and dilution plating quantification. MSSA was significantly reduced on the MP compared to smooth surface (Additional file 1: Table S1) with this recovery method. Microscopy methods were utilized to ensure that the RODAC agar efficiently removed bacteria from the MP and smooth surfaces. Scanning electron microscopy (SEM) surface examination indicated that before RODAC recovery, the smooth silicone surface had extensive contamination compared to the MP surface (Additional file 2: Figure S1A and B). After RODAC recovery, both surfaces were without visible contamination (Additional file 2: Figure S1C and D). These results are consistent with quantitative results from the immersion assay (Figure 2).

### Transfer and persistence of bacteria on surfaces

**Figure 4:**
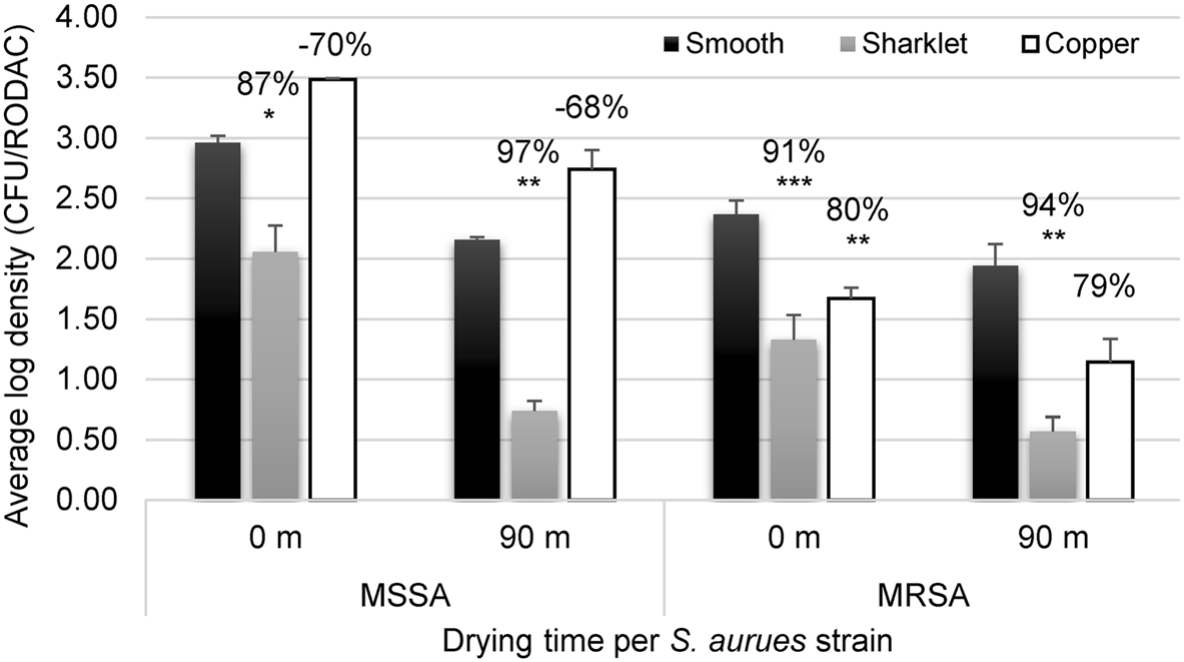
**Comparison of Sharklet MP to Copper antimicrobial surface.** MSSA and MRSA were used to challenge smooth unpatterned film, the MP film, and copper foil (99.9% pure) using a touch incident with time points sampled after 0 and 90 m of drying. Average log density values are presented for smooth, the MP, and copper surfaces. Error bars represent the SEM for 3 independent experiments. The percent reduction values were calculated using individual log reduction values comparing either Sharklet MP or copper to smooth control samples. *p <* 0.05 (*), *p <* 0.01 (**), *p <* 0.005 (***).

Both MSSA and MRSA on a surfaces were evaluated using the combination transfer and persistence assay with RODAC recovery methods. Transfer of MSSA onto the MP acrylic film was reduced 87% (LR = 0.90; *p* < 0.05) compared to smooth film (Figure 4). MSSA persistence on the MP was further reduced 97% (LR = 1.54; *p* < 0.001) after 90 m of drying (Figure 4). ANOVA analysis and Tukey grouping identified that reduction of MSSA after 0 m (transfer) grouped significantly differently (*p* < 0.05) than its reductions seen after 90 m of drying (survival). The MP reduced MRSA compared to smooth controls by 91% (LR = 1.04; *p* < 0.005) after 0 m and 94% (LR = 1.26; *p* < 0.005) after 90 m of drying (Figure 4).

Antimicrobial copper, which is marketed for its ability to reduce environmental contamination [23,44], was not effective at reducing MSSA contamination compared to smooth acrylic film after 0 m or 90 m. Copper did reduce MRSA by 80% (LR = 0.70; *p* < 0.002) after 0 m and 79% (LR = 0.69; *ns*) after 90 m of drying compared to smooth controls (Figure 4). Importantly, the MP reductions in MSSA contamination grouped in statistically higher log reduction groups from that of antimicrobial copper using Tukey post-test ANOVA analysis. These data demonstrated that the MP was more effective than antimicrobial copper surfaces in limiting bacterial contamination transfer and survival in the touch transfer assay.

## Discussion

The MP consistently demonstrated a reduction in microbial attachment, transference, and survival following simulated real-world inoculation methods. While the initial phase of this study identified the MP’s ability to limit bacterial attachment of MSSA and MRSA (Figure 2), high-touch surfaces in the environment are commonly contaminated by touch and sneeze-like events and not complete immersion. Additionally, the MP has potential to limit transference and persistence of bacteria, but the immersion method did not allow evaluation of those individual events. Therefore methods were further developed to simulate real-world inoculation events. Spray inoculation allowed for uniform and reproducible loading of inoculum onto surfaces to evaluate bacterial persistence over time (30–90 m) and touch transference assays were used to mimic indirect bacterial spread on high-touch surfaces through transfer alone therefore those assays required sampling after 0 m. Bacterial loads were sampled from the MP or smooth surfaces using RODAC contact plates [5,45,46]. The RODAC plates were used to quantify remaining bacterial loads, which proved to be a reproducible method which is not commonly used in standardized test methods [36,47,48]. Validation of RODAC sampling efficacy was done qualitatively using SEM (Additional file 2: Figure S1) and quantitatively using previously-optimized ultra-sonication (Additional file 1: Table S1) [34,41,49,50]. These data substantiate the use of RODAC contact sampling to test the bacterial load present after inoculation and drying on both the MP and smooth surfaces. Importantly, the MP demonstrated reduced bacterial contamination regardless of the inoculation and sampling method.

Interestingly, significantly superior reductions in bacterial load of MSSA on the MP after both transfer and survival time points compared to transfer alone were identified using Tukey grouping analysis (Figure 4; *p* < 0.05). This suggests that independent mechanisms are limiting bacterial transfer as well as bacterial survival after interaction with the MP surface. This report is the first to demonstrate that use of a microtopography can result in accelerated loss of bacterial viability compared to a smooth surface (Additional file 1: Table S1). Loss of bacterial viability following reduced bacterial surface interaction is not surprising since microbial transition to a tolerant sessile physiology relies heavily on surface adherence [51,52]. The MP was engineered and optimized to achieve specific surface energies, which reduce bacterial interaction and attachment compared to a smooth surface [29,53]. Therefore, the inability for bacteria to efficiently adhere to the MP is potentially responsible for the loss of bacterial viability in addition to the limited initial transfer of bacteria to the MP.

Antimicrobial copper has been the most popularly implemented surface technology able to demonstrate a reduction in bacterial contamination in both laboratory and clinical environmental testing [23,44]. Therefore, in this study, antimicrobial copper was compared to the MP, and the MP outperformed antimicrobial copper in reduction of bacterial transfer and survival. Copper was ineffective in limiting MSSA, while the MP reduced MSSA by up to 97% (p < 0.05) when compared to smooth acrylic control surfaces. Copper demonstrated 80% (p < 0.002) reduction of MRSA as compared to 94% (p < 0.005) reduction with the MP (Figure 4). The finding that the MP was more effective at limiting bacterial load 90 m after inoculation is intriguing. While the MP surface limits initial transfer due to surface energy changes [53,54] as well as perceived persistence of organisms, the copper surfaces appear to only limit persistence based on cytotoxic effects occurring after longer durations. The fact that the MP does not require cytotoxic compounds or leaching chemicals to be an effective alternative to traditional antimicrobials such as copper for limiting bacterial contamination is a distinct advantage.

The impact of these results is highly relevant given the evidence linking surface contamination to nosocomial infections [5,11,55,56]. Survival of *S. aureus* (including MRSA) on dry inanimate surfaces can range from 7 days to 7 months [8]. The existing and emerging surface decontamination and cleaning methodologies were clearly evaluated in a recent review by Weber and Rutala [22]. They discussed advantages and disadvantages of many hygiene practices and contamination resistant surfaces including the MP. Unfortunately, they found that while education and improved hygiene practices would theoretically contribute to fewer HAIs, little positive effects have been observed. Therefore, a technology that limits contamination regardless of human error is warranted. Additionally, chemical antimicrobial applications can also be problematic to vulnerable patient populations including neonates and young children and are often avoided. Wide implementation of antimicrobial surface technologies with direct kill mechanisms are concerning due to their potential to provide selective pressure for resistant micro-organisms. Heavy metal resistance has already been identified with clinically-relevant bacterial species showing resistance to silver [24,57,58] and copper [59]. The MSSA strain tested in this study exhibited tolerance to copper surfaces for 90 m but not to the MP. Considering that antimicrobial copper surfaces have been shown to reduce HAI rates combined with MRSA or VRE colonization when implemented in ICU rooms [23] and the MP outperformed copper when testing transfer and survival of MSSA and MRSA *in vitro*, this study suggests that the MP may help reduce infection rates and improve patient care. Continued testing of the MP surfaces in clinical settings should provide further evidence of the nature and magnitude of the benefits to patients.

## Conclusion

The MP surface is an effective and attractive method to broadly reduce microbial contamination on surfaces without the use of antimicrobial agents. The studies presented here clearly demonstrate that the MP reduces microbial transfer and when compared to the same material without the MP present. When adopted into real-world use, application of the MP onto high-touch surfaces in hospitals or shared public spaces is expected to limit environmental contamination of infectious microorganisms. Given that preliminary clinical evidence exists that antimicrobial copper implementation in hospital rooms decreases HAI rate, similar implementation of the MP, which outperformed copper in the transfer and persistence *in vitro* study, has potential to reduce the incidence of HAIs.

## Competing interests

All authors, except ABB, completed work while being employed by Sharklet Technologies, Inc. ABB is a paid consultant of Sharklet Technologies, Inc.

## Authors’ contributions

All authors had significant contributions to the science discussed. EEM, DM, RM Mettetal, RM May, EMD, KKC, ABB, and STR all contributed to the design and objectives of experimental analysis. EEM, DM, MR Mettetal, and RM May combined to carry out testing. All authors read and approved the final manuscript.

## Supplementary Material

Additional file 1: Table S1 Quantification of bacterial persistence using dilution plating. MSSA was aerosolized onto smooth or the MP acrylic film and allowed to dry for 90 m. 8 mm biopsy punches were used to cut film samples to suspend bacteria and dilution plate. Smooth and MP associated log densities with resulting log reductions are presented along with the *p* value using a single paired t-Test. Click here for file

Additional file 2: Figure S1 MSSA contamination persistence recovery. 1 × 10^7^ CFU/mL was prepared to immerse smooth and the MP surfaces. Samples immersed in a suspension of MSSA were rinse 3 times, sampled, and then prepared for SEM imaging. Smooth surface before RODAC **(A)** and Sharklet MP before RODAC sampling **(B)** are pictured adjacent to images after RODAC sampling **(C and D)**. Click here for file
