# Supplementary material for: Surface micropattern limits bacterial contamination
Source: Antimicrob Resist Infect Control. 2014 Sep 17;3:28. doi: 10.1186/2047-2994-3-28 (PMC4166016; doi:10.1186/2047-2994-3-28)
Supplement: Additional file 1: Table S1 — Quantification of bacterial persistence using dilution plating. MSSA was aerosolized onto smooth or the MP acrylic film and allowed to dry for 90 m. 8 mm biopsy punches were used to cut film samples to suspend bacteria and dilution plate. Smooth and MP associated log densities with resulting log reductions are presented along with the p value using a single paired t-Test. [file 2047-2994-3-28-S1.docx]

**Table S1.** Quantification of bacterial persistence using dilution plating. MSSA was aerosolized onto smooth or the MP acrylic film and allowed to dry for 90 m. 8mm biopsy punches were used to cut film samples to suspend bacteria and dilution plate. Smooth and MP associated log densities with resulting log reductions are presented along with the *p* value using a single paired t-Test.

| **Organism** | **Material** | **Drying**  **Time**  **(min)** | **Sampling Method** | **Smooth**  **Log**  **Density** | **Sharklet**  **Log**  **Density** | **Log Reduction** | **Percent**  **Reduction** | ***p* value** |
| --- | --- | --- | --- | --- | --- | --- | --- | --- |
| MSSA | Acrylic  Film | 90 | Biopsy,  Sonicate,  Dilution plate | 4.14 | 3.75 | 0.36 | 56% | 0.028 |
|  |  |  |  | 4.16 | 3.71 |  |  |  |
|  |  |  |  | 4.16 | 3.92 |  |  |  |
