# Supplementary figures and images for: Surface micropattern limits bacterial contamination
Source: Antimicrob Resist Infect Control. 2014 Sep 17;3:28. doi: 10.1186/2047-2994-3-28 (PMC4166016; doi:10.1186/2047-2994-3-28)

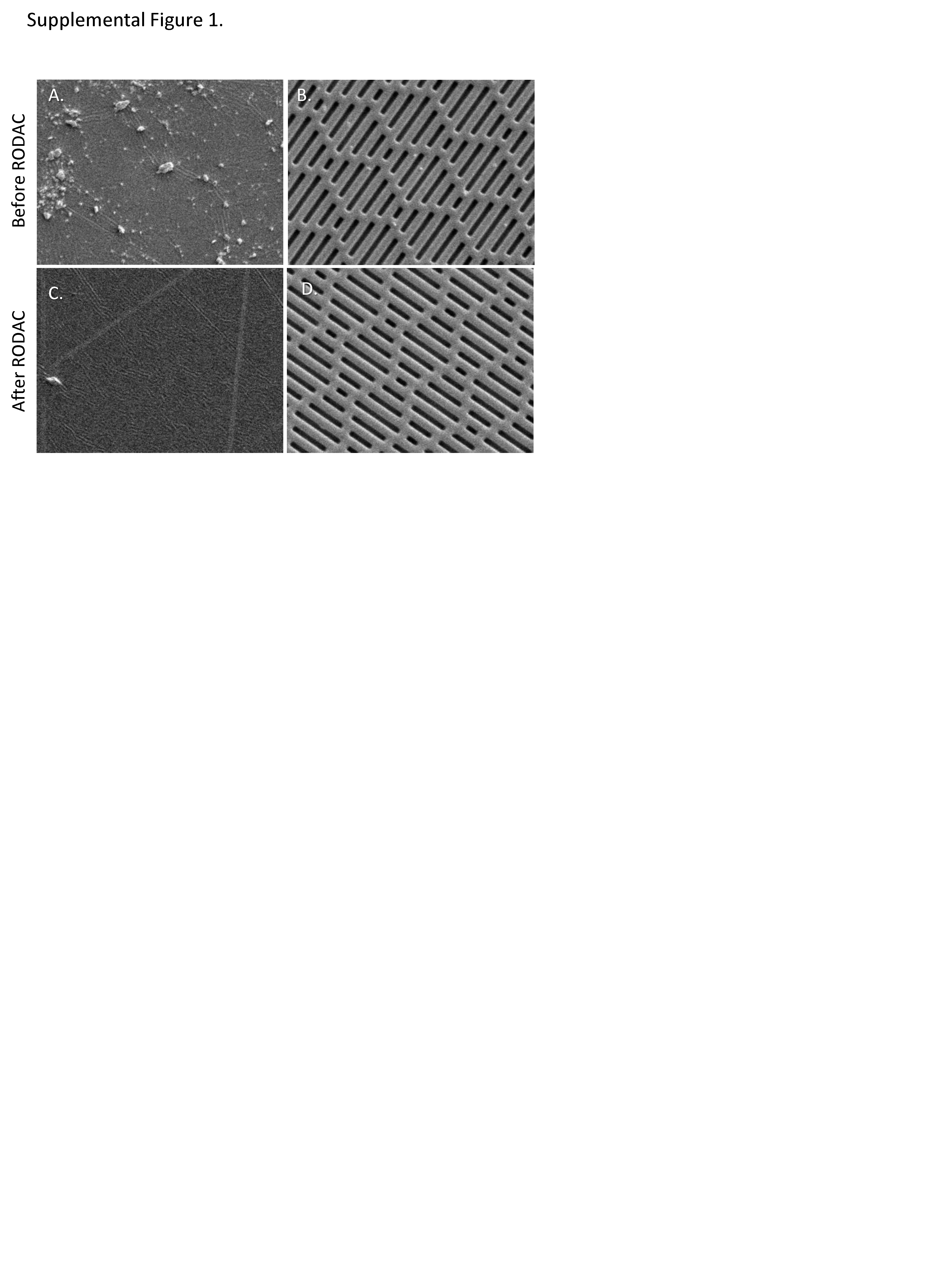

Supplement: Additional file 2: Figure S1 — MSSA contamination persistence recovery. 1 × 10^7^ CFU/mL was prepared to immerse smooth and the MP surfaces. Samples immersed in a suspension of MSSA were rinse 3 times, sampled, and then prepared for SEM imaging. Smooth surface before RODAC (A) and Sharklet MP before RODAC sampling (B) are pictured adjacent to images after RODAC sampling (C and D). [file 2047-2994-3-28-S2.tiff]
